# FaXToR: the hard X-ray micro-tomography beamline at the Spanish synchrotron ALBA

**DOI:** 10.1107/S160057752500997X

**Published:** 2026-01-01

**Authors:** Alessandra Patera, Federico Cova, Victorien Bouffetier, Llibert Ribo’ Mor, Steven Wohl, Gabriel Vicent Jover Manas, Caori Organista, Josep Nicolas, Alberto Mittone

**Affiliations:** a Alba Synchrotron Light Source Carrer de la Llum 2-26 08290Cerdanyola del Valles Catalonia Spain; b Argonne National Laboratory 9700 S. Cass Ave Lemont IL60439 USA; RIKEN SPring-8 Center, Japan

**Keywords:** micro-tomography, biomedicine, paleontology, material science

## Abstract

FaXToR is the first hard X-ray imaging beamline built and commissioned at the Spanish synchrotron light source ALBA, and therefore the start of user operation marks the introduction of a new imaging user community to the facility. FaXToR has pushed the implementation of a multi-scale, multimodal approach to investigate the morpho-structural changes of a number of materials within a range of applications, including biomedicine, material science, cultural heritage and paleontology. In this work, we describe the FaXToR beamline optical layout and the beam properties and we provide details on the flexible in-house design of the endstation. Beamline commissioning results are presented as well. Finally, an overview of its main applications is provided, defining the main strategic program the beamline will develop during its lifetime.

## Abstract 2

The Spanish synchrotron ALBA has recently inaugurated BL31-FaXToR, a beamline for hard X-ray micro-tomography. The design, performance and scientific program of the beamline are described.

## 1. An overview of the new BL31-FaXToR beamline

The FaXToR (FAst X-ray TOmography and Radioscopy) beamline (BL) at ALBA synchrotron is dedicated to fast micro-tomography in the hard X-ray regime range. The BL, located at port 31, is fed by a short in-vacuum multipole wiggler (Marcos *et al.*, 2023 ▸). Both filtered white and monochromatic beams are available at the endstation, with energies ranging from 10 to 70 keV (depending on the operation modes). The maximum beam size is 41 mm × 16 mm (H × V) at the lead screen on the experimental hutch, located downstream in the endstation (at 41.29 m from the source). The endstation presents a versatile design, enabling the possibility of multi-scale studies due to its pool of detection systems which make it possible to image one sample quasi-simultaneously at different spatial and temporal resolutions (Mittone *et al.*, 2022 ▸). *In situ* devices, such as compression rigs, climatic chambers, cryo chambers, *etc*. are also foreseen. Similarly, multimodal imaging will be enabled with the integration of a grating interferometer setup, which will provide different information about the sample (*i.e.* differential phase-contrast and scattering/dark field) (Weitkamp *et al.*, 2005 ▸), with an increase of sensitivity to low density materials at a reduced radiation dose. Such implementation targets a restricted number of users in the different fields, opening the possibility, for example, to quantify water content during hygroscopic loading in porous media or to investigate very low absorbing and micrometre scale features in a number of pathologies.

The beamline is designed to sustain high data throughput experiments, as well as dynamical studies with a time resolution of the order of sub-second (for full tomography), which requires on-the-fly 3D tomographic reconstruction and visualization pipelines.

The beamline aims to serve a broad user and industrial community, spanning medical and pharmaceutical applications, materials science, geology, paleontology and cultural heritage.

Particularly focused on life science and pre-clinical applications, FaXToR user’s program foresees its involvement in the following dedicated areas:

*Tissue imaging.* The cases of interest are: neuro-imaging (Bosch *et al.*, 2022 ▸), gynecological diseases, liver cirrhosis and its alteration with therapies (Wagner *et al.*, 2021 ▸), bone and cartilage pathologies (Turunen *et al.*, 2020 ▸), cardiovascular changes (Planinc *et al.*, 2021 ▸) and lung-imaging (Frank *et al.*, 2022 ▸).

*Metal-based theragnostic*. Metal nanoparticles are clinically used for diagnostic and therapeutic applications. However, quantifying their 3D distribution within tissues is challenging due to their small diameter. By exploiting their electron density and X-ray attenuation at a synchrotron, the biodistribution characterization of different types of nanoparticles in soft tissue has been demonstrated (Cörek *et al.*, 2020 ▸).

*Food and agriculture*. The internal features and compositions of plants, seeds, soil and food in a quick and non-destructive way to enhance their use, conservation and productivity and to understand the 3D inner structures of raw and processed food products thus promoting food safety and security (Indore *et al.*, 2022 ▸).

*Geoscience.*X-ray imaging enables the non-destructive examination of geological samples, allowing for the visualization and characterization of fractures, their geometry, and their mineralogical composition. The dynamic imaging capabilities of the BL enable 2D study of fracture propagation and deformation processes in volcanic rocks in real time during *in situ* experiments. Real-time observations can shed light on the mechanisms that trigger volcanic eruptions and the factors contributing to the propagation of fractures (Okumura *et al.*, 2013 ▸).

*Energy storage.* FaXToR’s relatively large field of view (FOV) will capture dynamic processes such as the evolution of lithium-ion distribution, electrode morphology changes, and the formation of solid–electrolyte interfaces in real time during charging and discharging cycles within full sized lithium batteries while zooming in particular regions of interest simultaneously (Abrego-Martinez *et al.*, 2023 ▸). The 3D imaging techniques available at the beamline can also help with understanding the process of dendrite growth and formation, which is a significant concern for safety reasons. The study of the development of dendrites can help to design strategies to mitigate their impact, ultimately enhancing the safety and longevity of lithium batteries. Another important subject that can be studied at FaXToR is the thermal runaway of commercial batteries.

*Metal foams and structural materials.* Metal foams have emerged as a promising class of materials with diverse applications ranging from lightweight structural components to efficient heat exchangers (Kamm *et al.*, 2021 ▸). Users at the BL could capture the porosity and fluid flow inside the foams, the process of formation of the metallic foams when using manufacturing processes such as gas injection into molten metals. *In situ* studies can also be performed to evaluate how different foams respond to external stimuli such as mechanical loads or thermal changes.

*Additive manufacturing.* FaXToR provides users with a high temporal resolution and FOV thus allowing the study of additive manufacturing *in operando* processes to gain insights into the relationship between material properties and printing parameters (Kenel *et al.*, 2017 ▸).

FaXToR is paving the way for the user community interested in hard X-ray imaging which will be further expanded in the near future (Perez *et al.*, 2022 ▸), enabling full multiscale sample characterization. In the next sections, we provide an overview of the BL layout and source properties, discuss the commissioning procedure and results, and finally describe our strategy for providing users with a multiscale experience at FaXToR.

## 2. Beamline layout and properties: design

### 2.1. In-vacuum multipole wiggler

**Figure 1:**
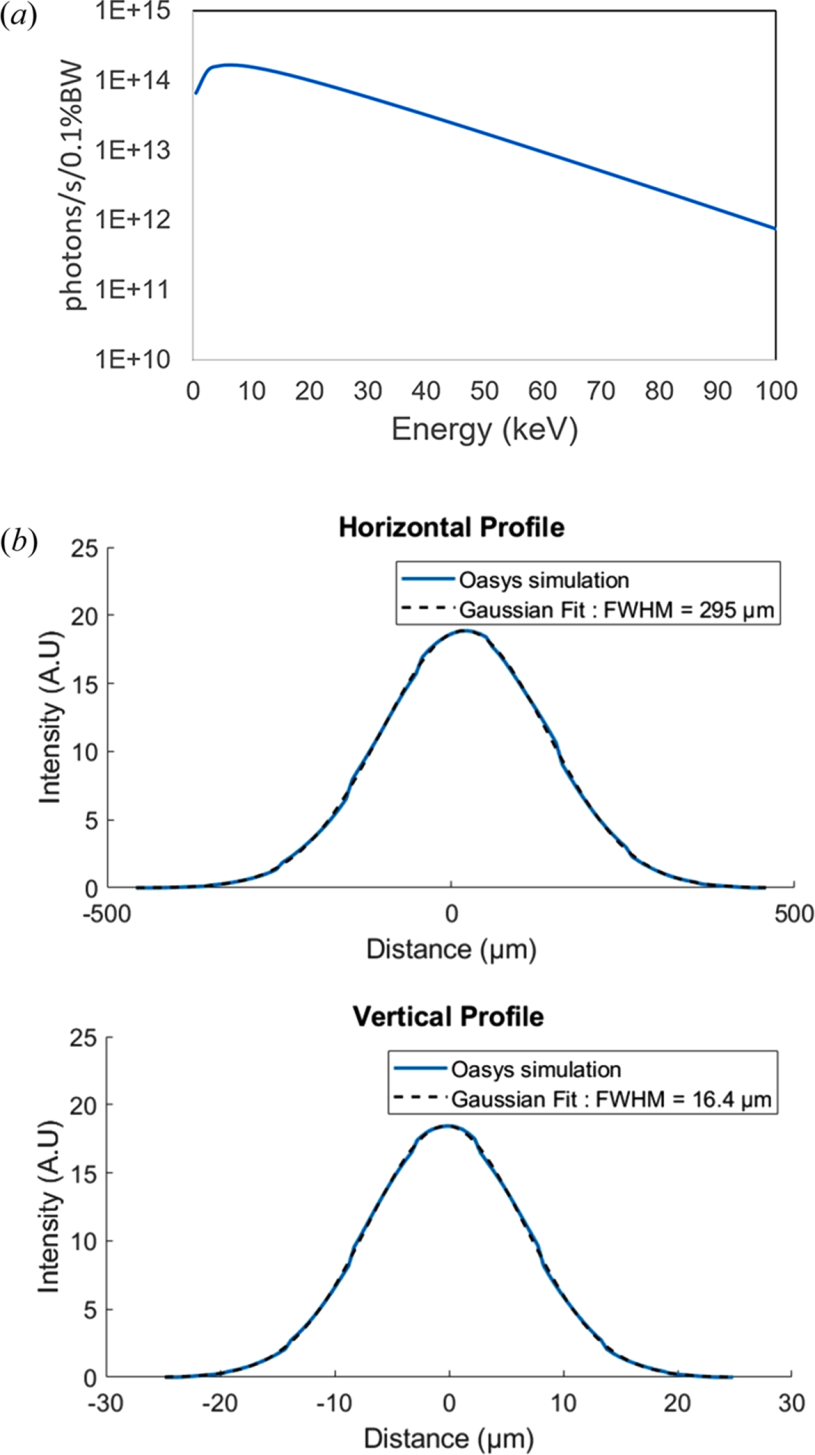
(*a*) Simulated spectrum, at an angular opening of 1 mrad × 0.4 mrad (H × V) and 250 mA of electron ring current. (*b*) Horizontal and vertical beam profiles expressed in FWHM.

The beamline is fed by an in-vacuum multipole wiggler (MPW) installed at port 31 of ALBA’s ring. The MPW is characterized by a critical energy of 14.7 keV and makes use of a 54 mm period with 11 full-size poles and a total length of the magnetic structure of 362.5 mm. The pole width is relatively small, in line with the small field region required over the whole gap range of 5–54 mm. The magnetic field strength peaks at 2.514 T at the minimum operational gap of 5 mm when the magnets have the minimum guaranteed remanence of 1.25 T. At this gap, the calculated source size (H × V) is 300 µm × 16 µm expressed in terms of full width at half-maximum (FWHM). The source size is largely influenced by the electron ring size, therefore FaXToR will largely benefit from a future reduced emittance of ALBA II. The calculated total power emitted by the MPW at 400 mA is 3.33 kW. Source size and power calculations have been performed using *OASYS* (Rebuffi & Sanchez del Rio, 2017 ▸) and *SPECTRA* (Tanaka, 2021 ▸) considering a gap of 5 mm. Currently ALBA runs in top-up mode at 250 mA; the use of 400 mA represents a conservative case and also in view of future machine current increase. Table 1 ▸ and Fig. 1 ▸ summarize the source properties. The front-end angular opening is set to 1 mrad × 0.4 mrad (H × V). The optics hutch is located at 17 m from the source.

**Figure 2:**
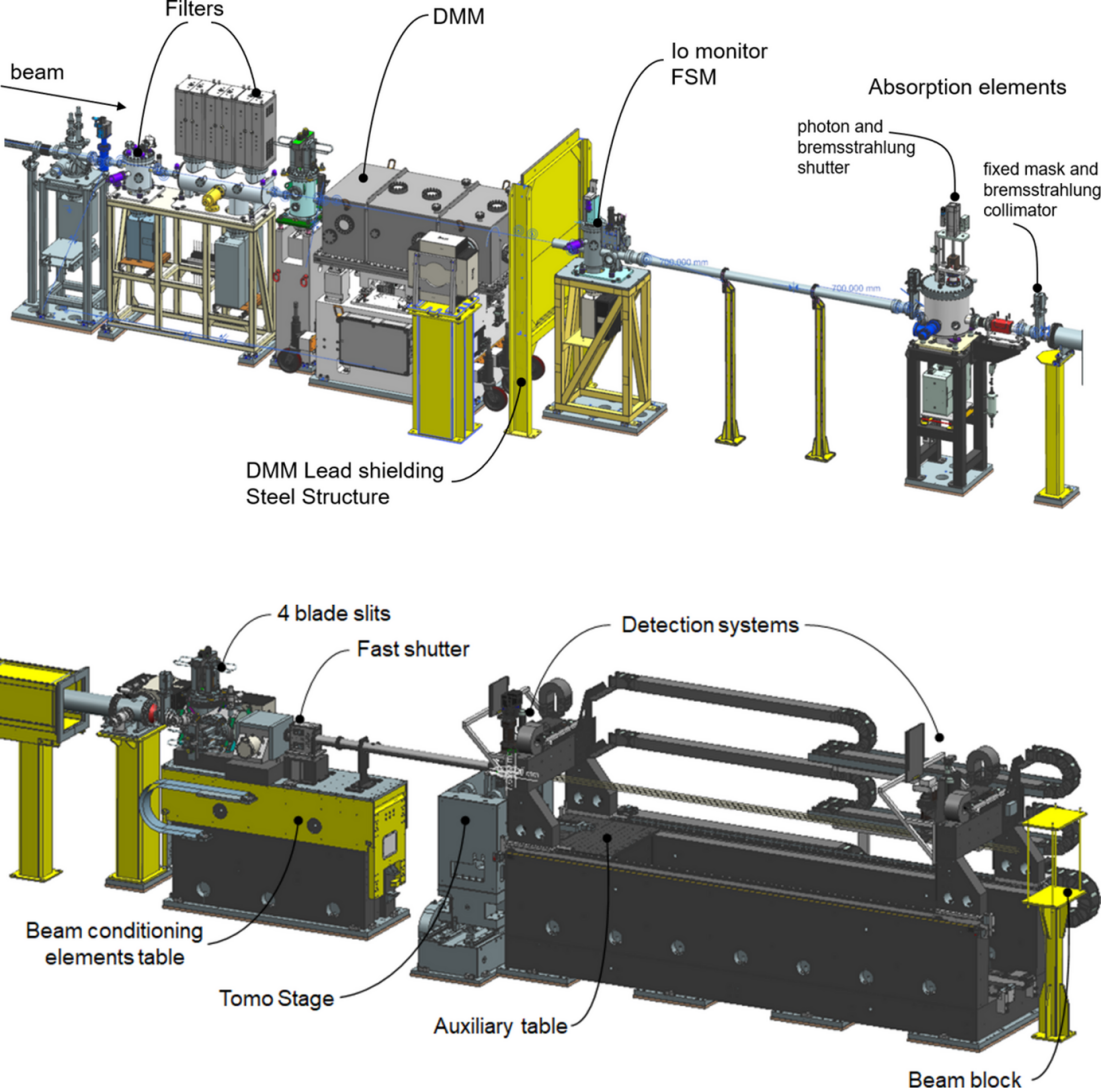
Diagrams of the FaXToR optics (top) and the endstation (bottom). The X-ray beam enters from the left side. The length of the optical hutch is 9.4 m while the experimental hutch total length is equal to 10.7 m.

The beamline specifications are listed in Table 2 ▸. A sketch of the beamline components is illustrated in Fig. 2 ▸.

### 2.2. Optics hutch elements

The main optical component is a double multilayer monochromator (DMM). It consists of two 500 mm long Si 〈100〉 crystals coated with two multilayer stripes, installed on a single pitch architecture in order to maximize its mechanical stability. Given the limited space available in the hutch, the DMM does not foresee a fixed exit, having a fixed longitudinal distance between crystals. The maximum vertical difference between monochromatic beams is 24 mm. More details are provided in the next paragraph. No other optics elements besides attenuators, vertical slits and diagnostics are included in the design of the optic hutch. As attenuators, two main filtering units are installed before the DMM to tune the X-ray spectra and minimize the power load on the downstream optical elements. They consist of a prefilter which is a water-cooled pyrolytic graphite foil of thickness 0.5 mm, and a multifilter unit which is composed of five movable racks where foils of different materials and thickness are mounted. The prefilter, installed before a 0.5 mm thick CVD diamond window, is used for a first power load attenuation and extra safety measure. All units are under ultra-high vacuum and located in separate vessels. The different filter foils in each rack of the multifilter unit can be independently positioned in front of the white beam by means of linear actuators. This offers the possibility of setting different filter combinations. They are supported on a water-cooled oxygen-free copper holder where the heat can be dissipated. In order to increase the thermal conductivity between the foils and the cooled support, some of the filters have been interfaced with an indium foil. Extensive finite element analysis (FEA) calculations have been carried out for the design’s optimization.

The diagnostic section is composed of a scintillator screen and an intensity monitor. The scintillator screen monitor is based on foils of CVD diamond and LuAG:Ce, for white and monochromatic beams, respectively. They can be switched by means of a motorized actuator. The emitted visible light is deviated by a reflector to a CCD camera that is used as diagnostic for beam commissioning purposes. Both foils are installed on a water-cooled oxygen-free copper absorber for thermal dissipation. As for the filtering unit, thermal contacts are critical for an optimal heat dissipation of the foil. The beam absorption equipment is placed after the diagnostics. It includes: a fixed mask, bremsstrahlung collimator and a photon and bremsstrahlung shutter, which is a copper water-cooled pad dimensioned to absorb the power of the impinging beam followed with a tungsten cube to stop the white beam radiation and protect the experimental hutch downstream.

#### 2.2.1. DMM design

The optical design of FaXToR was carried out using simulation tools included in the *OASYS* suite. In particular, *Shadow­Oui*, the *OASYS* add-on for ray-tracing based on Rebuffi & Sanchez del Rio (2016 ▸), was used to trace the rays and to evaluate the effects of the DMM crystals’ slope errors. Vibrations of the DMM play a pivotal role in terms of achievable performance of the beamline. The mechanical design is optimized for high vibrational stability, aiming at the highest frequency for the natural vibration modes of the system. In particular, the beamline is very sensitive to vibration modes that cause a pitch difference between the two mirrors, while it is quite forgiving to oscillations that preserve their relative pitch. For that reason, the DMM is designed in a way that the two mirrors share the same pitch stage.

The DMM is water-cooled and designed to withstand a maximum total power load of 700 W and power density <0.2 W mm^−2^. The design of the cooling system has been optimized via FEA calculations. Thermal dissipation between crystal and copper pipe is maximized by using indium–gallium based contacts.

The overall dimensions of the DMM and crystals are selected to meet space constraints while minimizing vertical beam size losses at higher energies, which are of the order of a few percent in the worst case (*i.e.* 50 keV). The multilayer (ML) crystal substrate measures 500 mm × 70 mm × 50 mm and has two coated surfaces, each 480 mm × 25 mm in size. One surface is coated with Ru/C layers, while the other is coated with W/B_4_C layers (single stripe). The calculated Bragg angle values are reported in Table 3 ▸. Expected beam sizes are shown in Table 4 ▸.

The choice of the coatings in terms of materials, periods and number of bilayers for the multilayer coated crystal is a balance in terms of reflectivity, energy bandwidth and beam footprint of the crystals.

Following this approach, as previously mentioned, the crystals are coated with two different stripes, Ru/C (*d*-spacing: 4 nm) and W/B_4_C (*d*-spacing: 2.5 nm) to cover the operational energy range [8, 50] keV. The energy bandwidth Δ*E*/*E* will vary in the range [2.6, 4]%. The optimization for the multilayer coating has been carried out using the *IMD* software integrated within *XOP* (Sanchez del Rio & Dejus, 2011 ▸).

Following the calculations, higher harmonic contamination has been found to be negligible (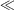
1%).

### 2.3. Experimental hutch components

The FaXToR experimental hutch is 10.7 m long and the first element is allocated at 32 m distance from the source. The hutch accommodates:

(*a*) a beam conditioning elements table, holding a set of four-blade water-cooled in-vacuum slits, a CVD diamond window, two fast shutters and a movable low vacuum pipe;

(*b*) a custom tomography tower (LabMotion, Belgium);

(*c*) a detection optics table, including an auxiliary table.

The conditioning table at the experimental hutch mostly hosts beam conditioning elements, in particular two fast shutters whose main role is to minimize the unwanted radiation exposure on the sample and other sensitive elements and to reduce the eventual motion artifacts linked to sample dynamics by means of an adequate synchronization with the acquisition pipeline. The first shutter, in-house made, is a non-periodic fast shutter based on the combination of two tungsten blades, each one driven by linear voice coil actuators. The blades synchronization achieves opening and closing times of 10 ms for a monochromatic beam size of 40 mm × 12 mm (H × V) aperture. The design provides flexibility to adjust the aperture dimensions and speed to be able to control the radiation dosage upon the sample, triggered by the image acquisition rate of the detector or timing device (TTL signal). The second shutter, currently under design, is foreseen to be integrated on top of the table. It is a shutter producing a periodic signal, triggered by the tomographic stage starting its spinning. It is based on a high-speed rotor which produces a signal frequency ranging from 100 to 1000 Hz, depending on the experimental conditions.

After the dual shutter system, a flying tube coupled with Kapton foil windows has been placed to minimize air absorption at lower X-ray energies and ozone production in filtered white beam conditions. The sample is positioned with respect to the incoming beam on an air bearing based custom system (Lab Motion, Belgium). According to the sample size and weight, the tomography stage can be used in two configurations. For samples of weight less than 10 kg, an *XY* micro-positioning stage is available on top of the rotary stage (LabMotion RT150ST), connected through slip rings with high precision and short range. However, in this configuration the rotation speed cannot exceed 300 rev min^−1^ depending on the sample mass. Whenever light samples at high speed (from 300 to 750 rev min^−1^) or heavy samples (from 10 to 20 kg) are used, the *XY* stage can be removed to satisfy the experimental needs.

Finally, the detector table is an in-house design that consists of two granite bridges mounted on a motorized rail and an auxiliary table. The bridges are designed to accommodate the two indirect detection microscopes. The microscopes make use of scintillator screens to convert X-rays into visible light and are coupled with CMOS cameras, as explained in Section 2.3.1. Furthermore, an auxiliary table is integrated to be positioned along the full detector longitudinal range and can be aligned in height according to the beam position. The auxiliary table is embedded in the detector table which can move independently of the detector positioners. This auxiliary table acts as a versatile multipurpose platform. It is compatible with custom sample environments and/or user-developed sample stages. Future upgrades exploiting the beamline capability will take advantage of the platform.

#### 2.3.1. Multiscale imaging capabilities

Two indirect detection optics, manufactured by Optique Peter, Lentilly, France, based on the combination of X-ray scintillation screens and visible optics lens systems are installed on the granite bridges located at the detection optics table. The first optics consists of a triple magnification system hosting 2×, 5× high resolution (HR) and 10× objective lenses which can be switched remotely according to the experimental needs. The second is a low magnification microscope (0.476×, 1×, 2.1×), optimized for experiments in which larger FOV and/or high-speed tomography are required. The bridges are installed on motorized rails allowing the propagation distances between the sample and the microscopes to be adjusted remotely. The possibility of using both microscopes simultaneously allowing multi-resolution imaging is foreseen thanks to the use of semi-transparent mirrors similar to the system reported by Mittone *et al.* (2020 ▸). LuAG:Ce type scintillator screens (CRYTUR, Turnov, Czech Republic) of different thicknesses are used. The thicknesses have been selected to match the optical specs of the microscope, in terms of depth of focus, to preserve the spatial resolution. Four different detection cameras are available at the BL and can be coupled with the two microscopes. Details are reported in Table 5 ▸.

Detectors send the data through frame grabbers in three data ingestion units (DIUs) located at the beamline. These DIU are directly connected to the Ultra Fast Storage (UFS) system at the High-Performance Computing (HPC) where both the raw and 3D reconstructed data are saved. The data can be remotely retrieved by the users following the ALBA policy for data transfer and storage. Different data processing workflows are being implemented as well as a user-friendly Python graphical user interface (GUI). This GUI allows users to easily choose methods from different libraries such as *TomoPy* (Gürsoy *et al.*, 2014 ▸) and *TomocuPy* (Nikitin, 2023 ▸), adjust the reconstruction parameters and visualize both intermediate and final results. The HPC provides a second workflow for on-the-fly (OTF) tomographic reconstruction that is able to provide more than three sets of three reconstructed slices per second. This workflow is based on the RECAST3D library (Buurlage *et al.*, 2018 ▸), and allows the user to select the three slices of interest to be shown during the real time visualization (Jover Mañas *et al.*, 2024 ▸). In addition, it is possible to perform data preprocessing, such as filtering, flat/dark field correction and a fast 3D low resolution tomographic reconstruction whenever high-speed imaging experiments are carried out.

To reduce the radiation damage on the detection system a continuous nitro­gen flow system is implemented at FaXToR optics. Avoiding the formation of a corrosive atmosphere minimizes the damage on internal reflective mirrors and on microscope objectives. Additionally, microscope objectives are radiation hardened (lead glass shielded, Optique Peter, Lentilly, France) in order to reduce lens browning, and consequent efficiency reduction, by X-rays. Finally, detection cameras are properly shielded with a lead envelope that prevents electronics damage, radiation induced noise and the consequent deterioration of the data quality.

## 3. Commissioning activities and main results

The commissioning of the beamline started in early 2024. The reported results within this work include:

(i) Power measurement strategy and results compared with simulations.

(ii) Commissioning of the source in terms of spectra and flux.

(iii) Monochromator calibration.

(iv) Properties of the white beam in terms of dose effects.

Using a water-cooled copper block to fully absorb the beam, the total power with different filter combinations was estimated by measuring the water temperature difference between the input and output of the water circuit. The resulting values were normalized to account for the difference in the ring current with respect to the simulation, and then both simulated and measured values were compared as shown in Fig. 3 of Patera *et al.* (2025 ▸). This set of measurements was used as reference for two scopes: on one side, to modulate the power before X-rays impinge into the DMM, depending on the experimental configuration and sample radiation sensitivity for a specific configuration; on the other side, to modulate the spectra around a peak energy, based on the experiment’s requests.

**Figure 3:**
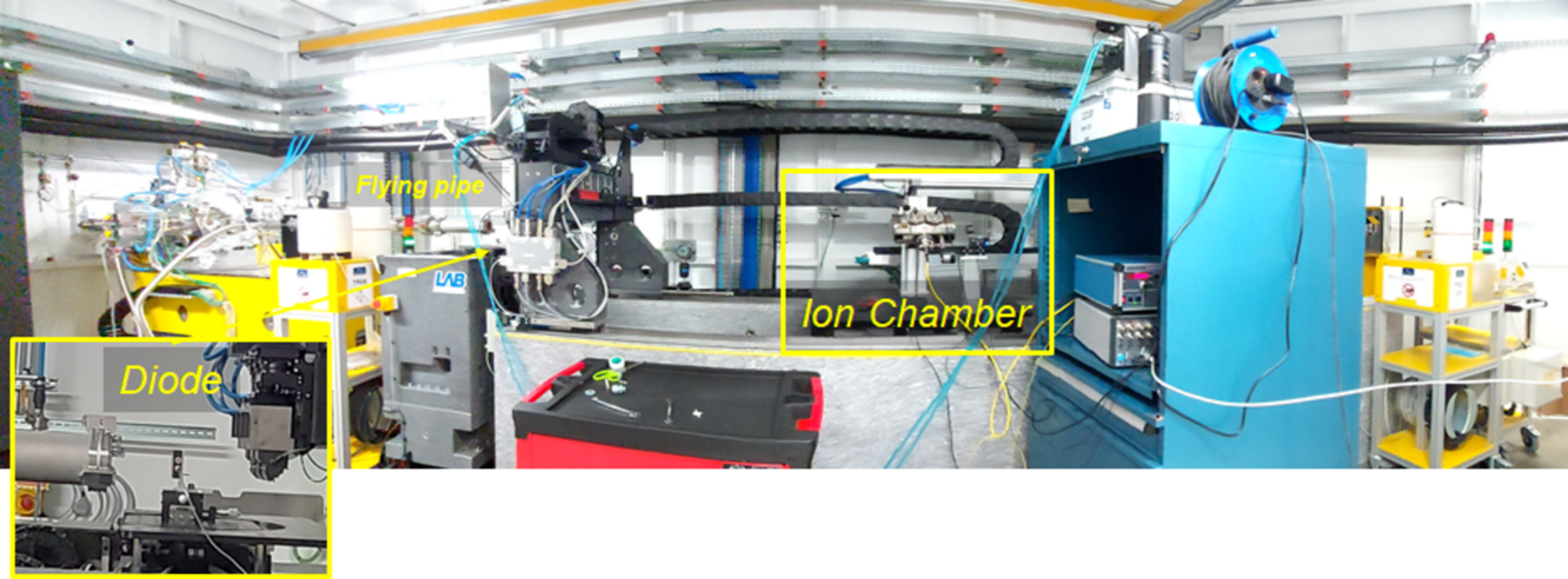
Commissioning settings in the endstation of the experimental hutch, including a Si diode on top of the tomostage, and in front of the detector to minimize the air scattering. For further calibration of the diode at energies above 20 keV, an ion chamber filled with 70% N_2_ and 30% Kr has been placed on top of the auxiliary table.

Source properties have also been characterized. In particular, the flux and the spectra have been measured under different conditions of filtering and beam modalities. For the intensity measurements, two measuring systems available at ALBA have been used (Fig. 3 ▸):

(*a*) An ion chamber, located on top of the auxiliary table, in which gas filling absorbs along with the monochromatic beam range of [8–50] keV. The distance between the fly pipe and the chamber is 42 mm.

(*b*) An Si diode, 10 mm thick (Cruz *et al.*, 2015 ▸), located on top of the sample stage, previously calibrated up to 22 keV energy. The distance between the fly pipe and the diode is 12 mm in this case.

**Figure 4:**
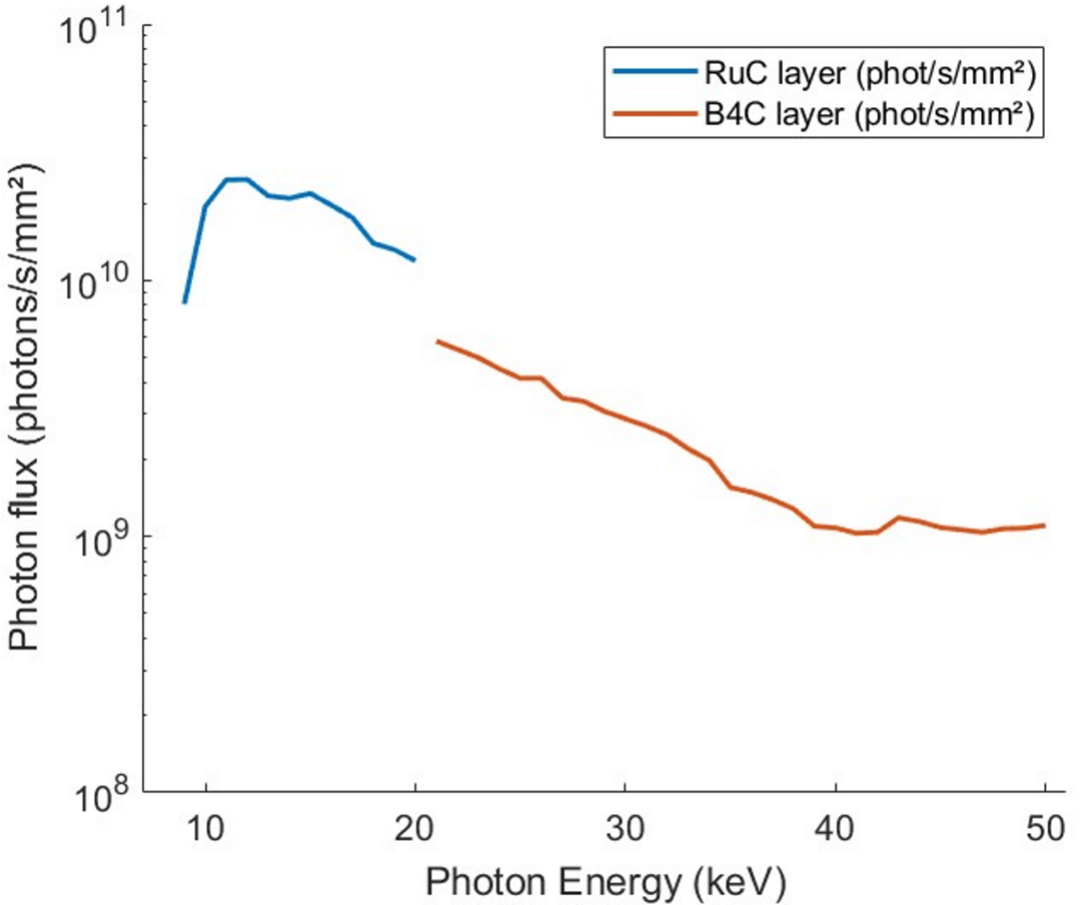
Comparison of the measured photon flux using a Si diode placed at the sample position when using the RuC mirrors of the monochromator (blue curve) and W/B_4_C mirrors (red curve). Filter combinations: 0.5 pyro + 1.1 CVD + 1C (the numbers before the symbols represent the material thickness in mm and the symbols correspond, respectively, to material type, *i.e.* pyrolithic graphite, diamond windows and carbon).

The flux result is plotted in Fig. 4 ▸, in both stripes.

**Figure 5:**
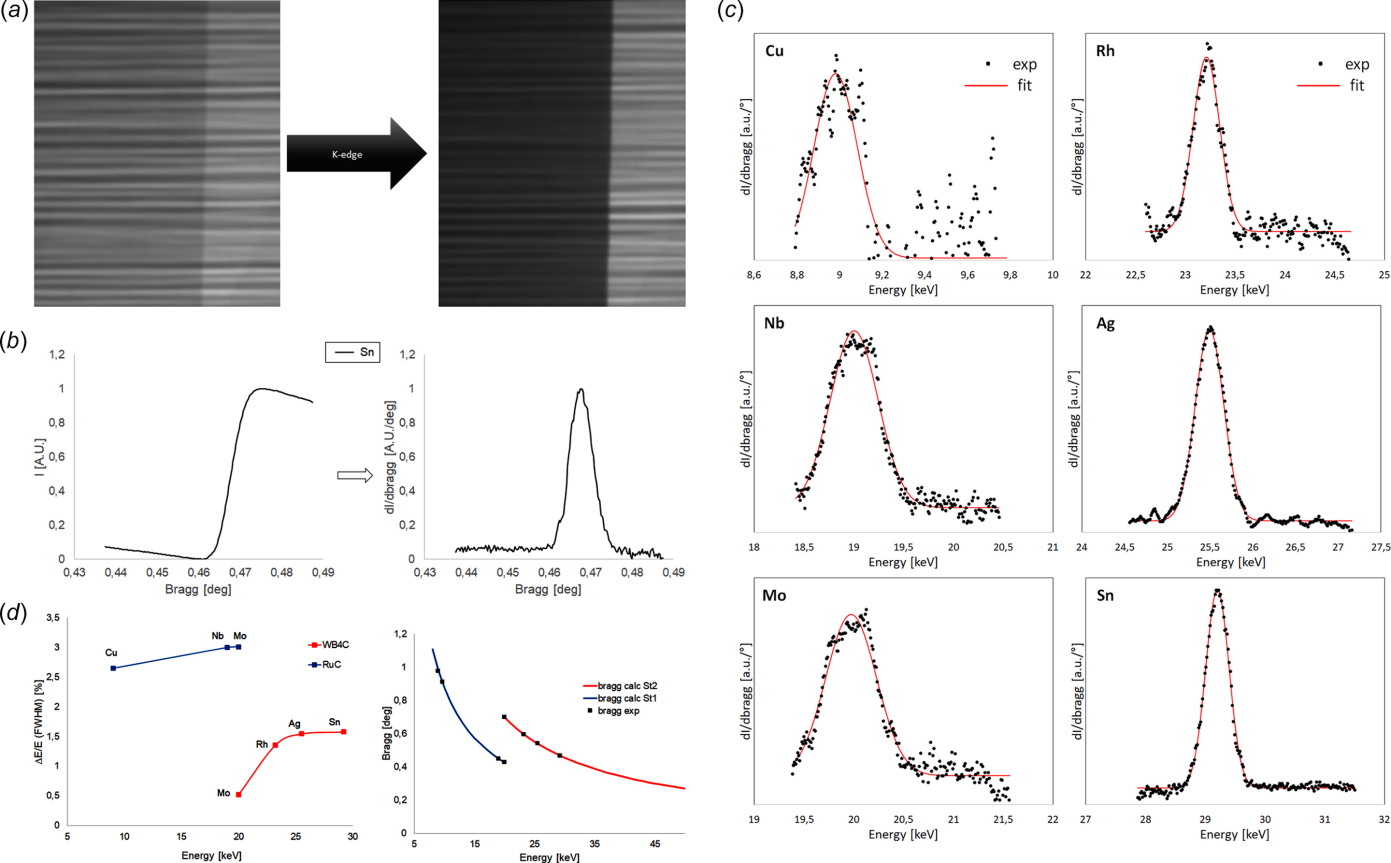
(*a*) Explanation of the procedure adopted to fine tuning the Bragg values from the radiographic measurements of the *K*-edge mask. The Bragg angle is scanned around the calculated values and the intensity values are measured into the detector plane. In the picture, an example with Sn foil (*E* = 29.2 keV) is shown. (*b*) The derivative of the intensity shows a peak around the corrected Bragg value. This procedure has been applied in both stripes (RuC on the left and WB_4_C on the right) and a correction factor has been applied. (*c*) Energy bandwidth calculations from the FWHM of the fitting function. The first value on the red curve representing the values on the second stripe is not considered. The energy values have been corrected after calibration of the zero angle of the monochromator with respect to the incident beam. (*d*) The energy bandwidth (left) and the Bragg/energy relationship (right).

The monochromator has been commissioned and fully characterized. Following equation (1) of Morawe (2018 ▸), the refraction corrected Bragg equation for each ML stripe with a defined *d*-spacing has been inserted into the Sardana Control system (Reszela *et al.*, 2017 ▸) to define energy/angle values. Such values are considered, in the commissioning phase, as input to define the Bragg pseudo-motor position based on the desired energy, which depends on the motion of the vertical downstream stage. The modified Bragg equation assumes ideal conditions, including perfect alignment of the multilayer stripes with respect to the incident X-ray beam. However, in practical scenarios, misalignments or small deviations in the orientation of the multilayer surfaces relative to the beam axis can result in discrepancies between theoretical predictions and actual energy delivery. To fine tune the Bragg values and define the energy bandwidth, six metallic calibration foils made of different elements, with absorption edges (*K*-edge) covering an energy range between 8 to 30 keV, have then been inserted into the beam path. Each metal was positioned such that it partially occluded the camera’s FOV, thereby allowing for simultaneous detection of both attenuated and unattenuated beam regions. High-resolution radiographies have been collected with the triple magnification microscope coupled with the pco.edge 4.2 detector and 5× magnification objective, yielding an effective pixel size of 1.3 µm. X-rays are converted into visible light by an LuAG:Ce scintillator of thickness 300 µm. The Bragg motor is moved to the expected position corresponding to the edge energy and then a scan is performed around that value (Fig. 5 ▸). For the Mo foil, since its *K*-edge falls in the threshold energy between the two stripes, two sets of scans were made, one for each stripe, allowing the number of experimental data points for the second stripe (B_4_C/W) to be increased. The resulting image data from these scans was analyzed by defining two regions of interest (RoIs): one within the area shadowed by the metal foil and another in a flat, foil-free portion of the beam footprint. The intensity ratio between these two RoIs was calculated as a function of Bragg pseudo motor position, effectively generating a transmission curve for each foil. As expected, these curves exhibited a sigmoidal behavior, corresponding to the transition across the absorption edge. Figs. 5 ▸(*a*) and 5(*b*) illustrate the curve corresponding to Sn. To quantitatively determine the Bragg pseudo motor position associated with the *K*-edge of each metal, the first derivative of each intensity curve was computed. The pseudo motor position corresponding to the peak of this derivative—representing the point of maximum slope on the sigmoidal curve—was taken as the experimental Bragg pseudo motor value for the respective *K*-edge. Additionally, the FWHM of the derivative peak was extracted and used as an estimate of the energy bandwidth of the monochromator at the corresponding energy, providing insight into the spectral resolution of each multilayer stripe. These Bragg values were then converted into theoretical energies. Also, in Fig. 5 ▸(*c*), the real values for the six *K*-edges are plotted to illustrate the existing shift between the calculated and theoretical energies. This curve can also be fitted to obtain the center of the peak and the FWHM. Using the set of experimentally obtained energy–Bragg pseudo motor pairs, the initial theoretical energy–position calibration curve was refined. This empirical correction correlates Bragg pseudo motor positions with actual beam energies, compensating for mechanical misalignments. The corrected calibration curve has been parameterized and integrated into the control system. The energy bandwidth and the corrected curve are presented in Fig. 5 ▸(*d*). Sparse data for energies >9.6 keV are present in the case of Cu foil. A potential explanation could be related to the presence of Lu material in the scintillator, which has three *L*-edges in the range 9–11 keV.

The capabilities of white beam at the sample position have been deployed in terms of maximum FOV and dose rate. These sets of measurements, performed in both filtered white beam and unfiltered beam (extreme conditions), are used as well to refine the thickness of the white beam shielding located downstream in the experimental hutch.

**Figure 6:**
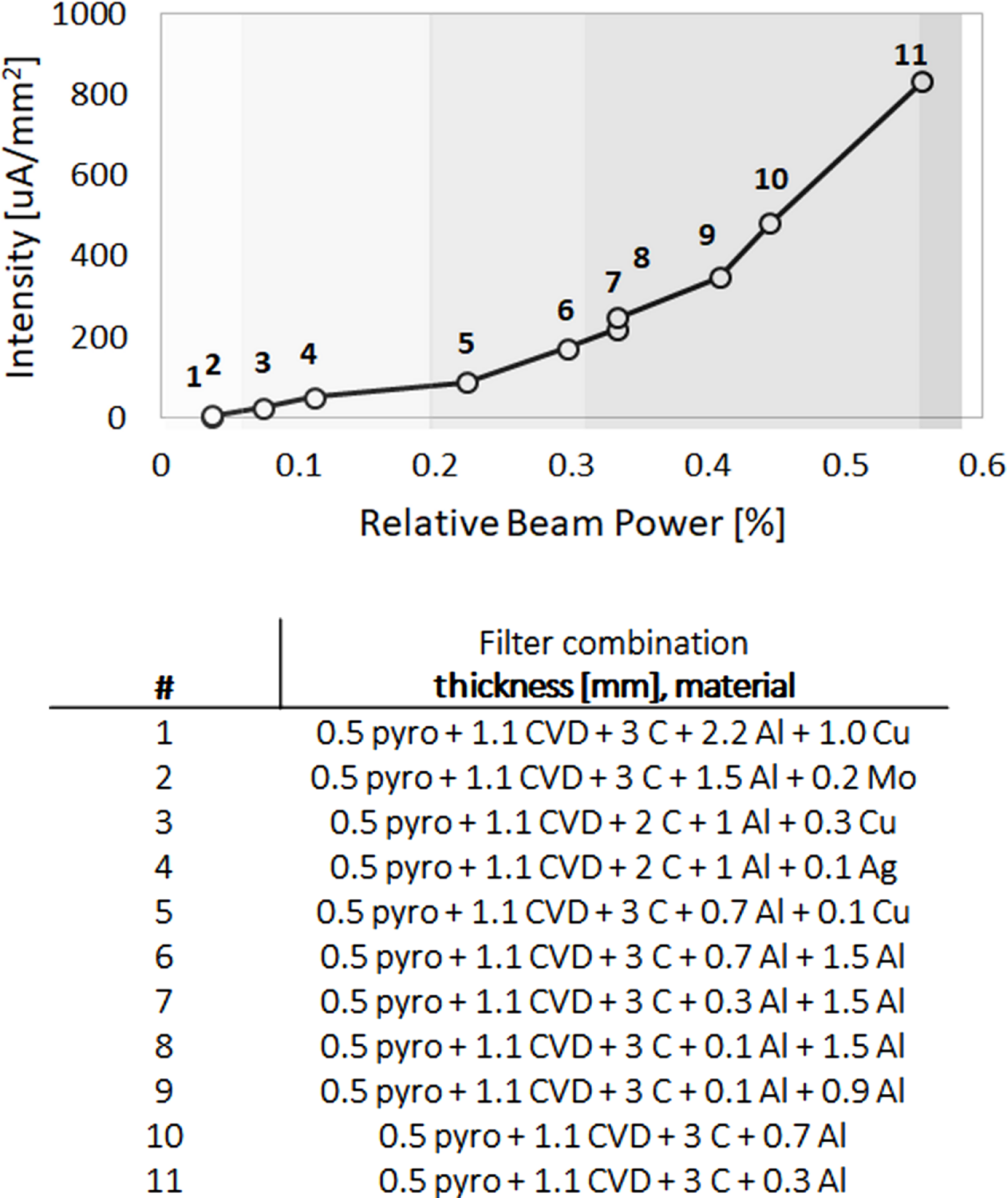
The plot on the left illustrates the current intensity of 1 mm^2^ filtered white beam, as measured with an Si diode in the experimental hutch. The filter combination is listed in the table below.

Starting from the MPW gap not fully closed (*i.e.* 20 mm) up to its minimum value and with movable masks in the frontend opening of 1.5 mm × 0.66 mm, the filter combination was selected in the multifilter units with a sequence capable of reducing 98% of the full power (unfiltered beam). The experimental hutch slits have been closed initially to a square area of 8 mm × 8 mm in order to safely bring the source gap into operational value (5 mm), then to a smallest squared area of 1 mm × 1 mm in order to collect the intensity signal on the Si diode with different filters combinations, up to the fraction of the beam which saturates the diode signal, corresponding to 55% of the incoming beam (combination #11) (Fig. 6 ▸).

Finally, extreme white beam conditions have been tested downstream in the experimental hutch, removing all non-static filters from the multifilter unit and fully opening the experimental hutch slits to let the entire beam pass through. The downstream block, composed of three 50 mm each layers of lead, one layer of 50 mm polyethyl­ene and one more layer of 50 mm lead, was sufficient to block photons in the worst-case scenario, at the moment considered as not realistic for user operation, resulting in a gamma dose rate of 0.1 µSv h^−1^, as measured on a dosimeter located outside the hutch along the beam direction.

## 4. First users: targeting the BL applications

In order to evaluate the performance of FaXToR, three fields of applications have been selected: biomedicine, paleontology and material science.

### 4.1. Biomedicine

**Figure 7:**
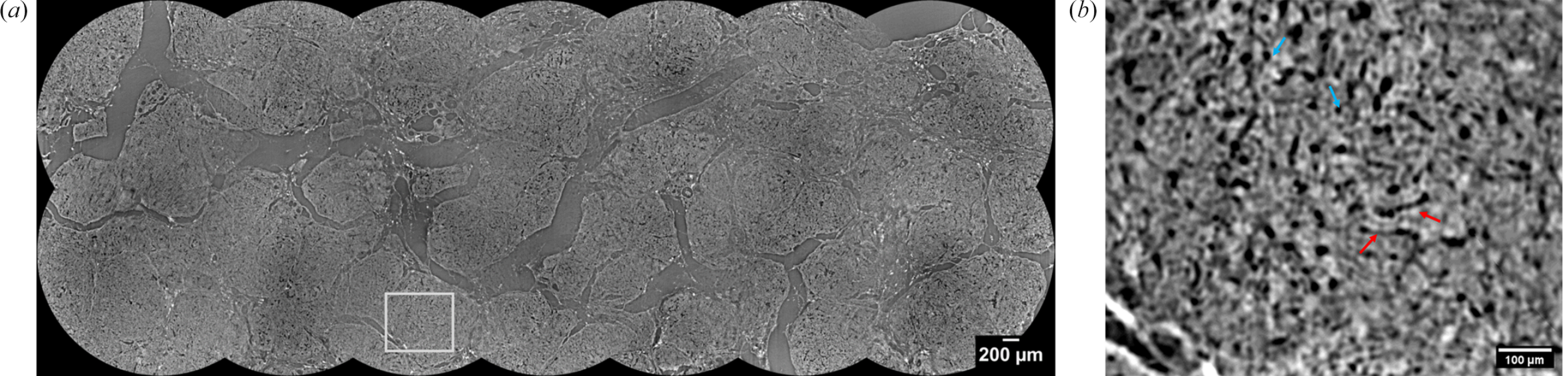
(*a*) 2D reconstructed stitched slide of 14 local tomographies from a region of cm-sized cirrhotic rat liver sample, showing an area of 8135 × 3062 pixels, resulting in 190 GB of 32-bit stitched local volumes. (*b*) Zoom on a ROI of 560 × 460 pixels, where key hepatic features are highlighted: sinusoids (blue arrows) and vasculature (red arrows).

In collaboration with scientists from Universitat Pompeu Fabra and Hospital Clínic de Barcelona, a total of six rat liver samples, embedded in a paraffin block of 30 mm × 20 mm, have been imaged to study the inner structural changes of rat liver in different pathological conditions. Each sample has been scanned with 0.65 µm isotropic voxel size resulting in coupling the pco.edge 4.2 detector and 10× magnification optics at 20 keV monochromatic beam energy. The sample to detector distance was optimized to 200 mm for enhanced contrast in tiny vessels and low dense compartment of the liver. In order to image a big portion of the whole cm-sized sample at high resolution, a protocol for automatic local tomographic acquisition, inspired by the work of Patera *et al.* (2021 ▸), has been implemented. Full-field reconstruction based on the NR Stitcher algorithm (Miettinen *et al.*, 2019 ▸) has been used to map the detailed morphological structure of the organs, optimizing and automating the data processing for FaXToR datasets, for which single tomography reconstruction was initially performed by applying the Paganin method for phase retrieval (Paganin *et al.*, 2002 ▸). Two thousand projections over 180°, plus 100 flat and 100 dark fields, have been acquired with exposure time of 75 ms. Fig. 7 ▸ shows a 2D reconstructed slice of rat liver with 14 regions merged with 20% of overlap.

### 4.2. Paleontology

**Figure 8:**
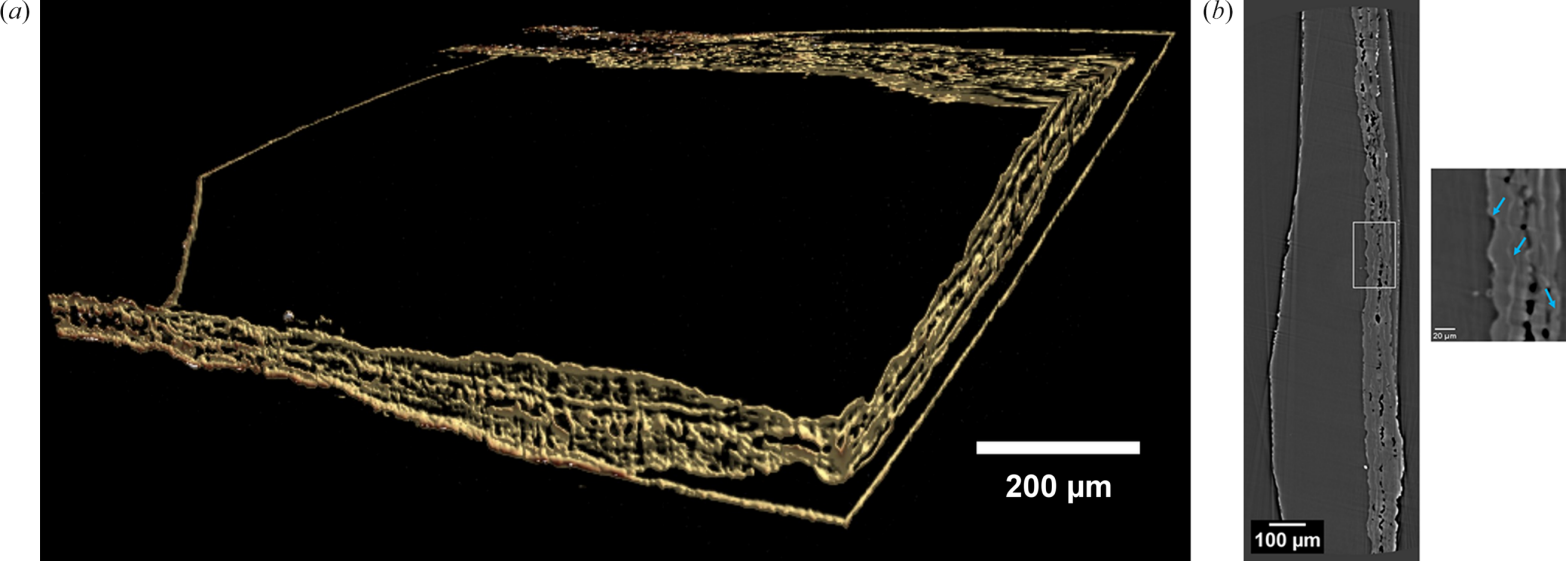
(*a*) Volume rendering and (*b*) 2D reconstructed cross section of a stem fragment of Frenelopsis, with dense parallel striation as highlighted by blue arrows in the ROI.

For paleontological samples, energies ranging from 30 to 50 keV are required to penetrate into the materials and produce good quality images from different sample features and thicknesses. A number of samples of angiosperms, or flowering plants, representing an abominable mystery for Darwin, have been scanned with an isotropic voxel size of 1.3 µm obtained by coupling the pco.edge 4.2 camera with the 5× HR objective. FaXToR is collaborating with scientists from the Universidad Autónoma de Madrid to unravel internal information, such as the microvascular structures, of the wetland fossils plants of Las Hoyas (Cuenca, Spain). A volume rendering showing a fragment of the stem and adpressed leaf of a 129 million years old extinct conifer, named Frenelopsis, is shown in Fig. 8 ▸.

### 4.3. Material science

**Figure 9:**
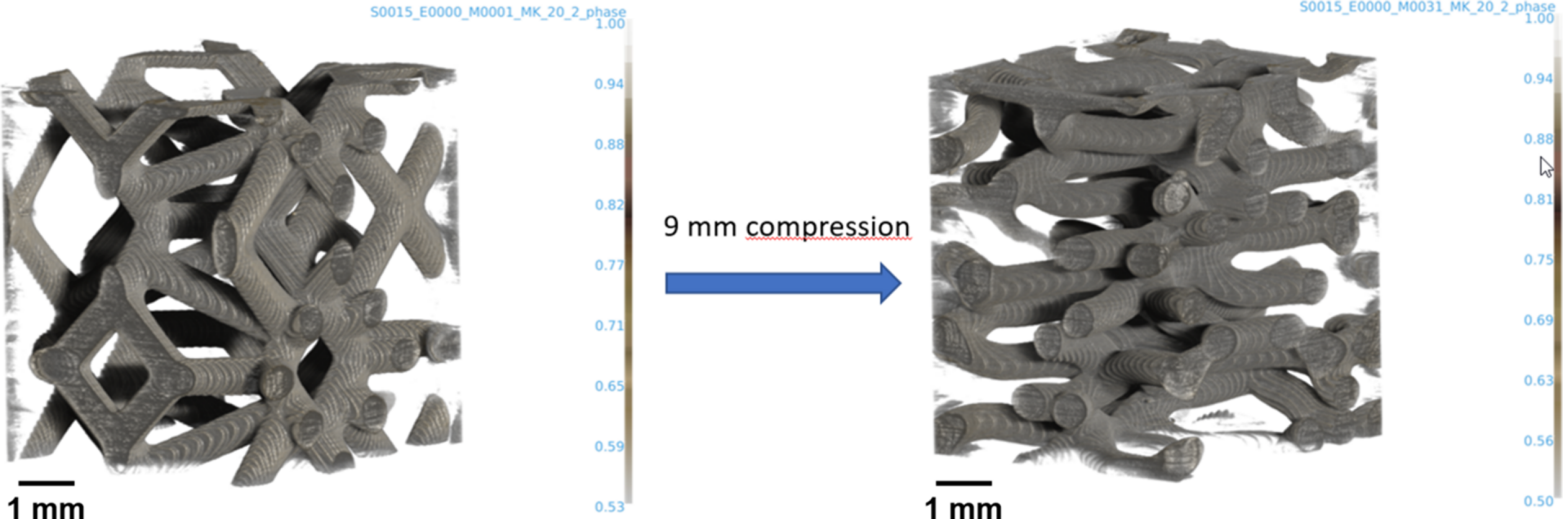
3D view of a quasi-static compression of a lattice-based elastomer. Tomograms were acquired at 17 keV and 3.25 µm pixel size.

In material science, the feasibility of the FaXToR fast imaging mode for *in situ* analysis of mechanical and morphological responses in open-structured architecturally designed metamaterials under high deformation rates has been assessed. Different samples designed and fabricated at the Norwegian University of Science and Technology (NTNU) by 3D-printed structures using additive manufacturing with controlled printing parameters have been tested at the BL. They are 10 mm cubes made of polymers, such as foams. The samples have been loaded in a compression rig, model CT5000 5kN from Deben Ltd, until failure. Full datasets, collected at different deformation rates, demonstrated the performance of the BL for the *in situ* characterization of materials. Fig. 9 ▸ shows 3D views of a quasi-static compression of a lattice-based elastomer.

## 5. Strategic user plan and discussion

Since the start of user operations, FaXToR has been demonstrating its capabilities in the strategic research field of ALBA: medicine, biology, paleontology, materials, environment and other related disciplines. Due to its versatility, the beamline provides a very powerful tool for a broad range of user communities. Furthermore, the start of its operation makes possible multiscale multimodal user programs foreseeing a correlation between morphological information obtained by X-ray micro-computed tomography with structural and chemical analysis as provided by other beamlines within ALBA.

While reinforcing the capabilities of the BL in spatial and temporal resolutions, along with the line of its commissioning and future upgrade plan, FaXToR aims at contributing and demonstrating the Spanish synchrotron uniqueness to be a one-stop shop for the different communities.

Finally, following the global trend, ALBA is foreseen being upgraded into a fourth-generation storage ring, under a project named ALBA II in the coming years (Perez *et al.*, 2022 ▸). Within the synchrotron upgrade plan, ALBA II will host two new imaging beamlines offering a nanometric sized X-ray beam for material science and bioimaging. In this context, FaXToR is paving the way for the user community interested in hard X-ray imaging which will be further expanded with the implementation of the new nano-probes, enabling full multiscale sample characterization.

## Figures and Tables

**Table 1:** Source parameters and corresponding values

Parameter	Value
Number of periods	5.5
Period length (mm)	54
Maximum field (T)	2.5
Minimum GAP (mm)	5
*K*	12.6
*E*_c_(0) (keV)	14.7

**Table 2:** FaXToR BL specification at the ALBA synchrotron for X-ray full-field radiography and tomography The maximum beam size is referred to on the sample plane, located at 36 m from the source. As seen in the energy spectra of the emitted photon flux [Fig. 1(*a*) ▸], imaging applications requiring highest flux will cover the range [10, 70] keV, when working in white beam mode. On the other side, due to the multilayer’s length, vertical beam lost is acceptable within the [8, 50] keV range when working in monochromatic beam mode.

X-ray source	Maximum beam size (mm)	Minimum pixel size (µm)	Beam modes	Techniques
In-vacuum wiggler	36 × 12	0.45	Filtered white beam	Absorption contrast
			Monochromatic beam	Phase contrast:
				Free-space propagation
				Grating interferometry

**Table 3:** Calculated coating performances and Bragg angles

Coating	Energy (keV)	Bragg angle (degrees)	Peak reflectivity	Δ*E*/*E* (%)
Ru/C	8	1.16	0.78	4
Ru/C	10	0.93	0.84	4
Ru/C	20	0.44	0.93	4.2
W/B_4_C	20	0.73	0.78	2.5
W/B_4_C	30	0.49	0.87	2.5
W/B_4_C	40	0.37	0.91	2.6
W/B_4_C	50	0.30	0.93	2.6

**Table 4:** Expected beam sizes in terms of FWHM (H size and V size) at 35.5 m from the source The calculated vertical size losses in % (V lost) are reported. In these calculations, V lost represents the difference between the vertical beam size considering an infinite long crystal and a finite (480 mm) crystal length.

Energy (keV)	H size 5 mm gap (mm)	V size (Ru/C, 4 nm) (mm)	V lost 5 mm gap (Ru/C, 4 nm)	V size (W/B_4_C, 2.5 nm) (mm)	V lost 5 mm gap (W/B_4_C, 2.5 nm)
8	34.89	12.53	0.00%	12.53	0.00%
10	35.02	11.33	0.00%	11.33	0.00%
20	34.74	6.36	18.83%	7.84	0.00%
30	34.80	4.24	32.54%	6.29	0.00%
40	34.95	3.18	41.58%	5.44	0.00%
50	34.91	2.54	47.21%	4.68	1.90%

**Table 5:** A list of detection components within the FaXToR portfolio

(1) Optic tower 1: triple magnification detection optics with three Mitutoyo objectives (2×, 5× HR and 10×)
(2) Optic tower 2: low magnification detection optics with a tandem Hasselblad objectives system (1×, 0.476/2.1×)
(3) Four cameras:
Detector 1: pco.edge 4.2 with 6.5 µm pixel size and 100 frames s^−1^ (Excelitas, Pennsylvania, USA)
Detector 2: pco.dimax-cs4 with 11 µm pixel size and 1102 frames s^−1^
Detector 3: C-Blue One Camera 7.1 MP with 4.5 µm pixel size and 207 frames s^−1^ (XIMEA GmbH, Münster, Germany)
Detector 4: Phantom S710 with 20 µm pixel size and 5k frames s^−1^ (Vision Research, New Jersey, USA)
